# Association of hydromyelia and acute compressive myelopathy caused by intervertebral disc extrusion in dogs

**DOI:** 10.1111/jvim.16433

**Published:** 2022-04-28

**Authors:** Philippa J. Johnson, Amy B. Todd‐Donato, Andrew D. Miller, Yu Wang, Chris Holm, Carolina I. Panisello‐Manterola, Claudia S. Colón Acevedo, Jonathan H. Wood

**Affiliations:** ^1^ Department of Clinical Sciences Cornell University College of Veterinary Medicine Ithaca New York USA; ^2^ Department of Biomedical Sciences Cornell University Ithaca New York USA

**Keywords:** canine, chronic, CSF, obstructive hydromyelia, syringohydromyelia

## Abstract

**Background:**

Hydromyelia is a common magnetic resonance imaging (MRI) finding associated with compressive myelopathy caused by intervertebral disc extrusion (IVDE).

**Objectives:**

To describe the MRI features of hydromyelia and explore its relationship to clinical history, neurological severity, and the duration of cord compression.

**Animals:**

Ninety‐one client‐owned dogs with a focal compressive myelopathy secondary to thoracolumbar IVDE.

**Methods:**

A retrospective observational study was conducted in which MRIs were blindly evaluated to grade and localize hydromyelia and measure the degree of spinal cord compression. Duration and severity of clinical signs were recorded. Differences between hydromyelia grades in these variables were statistically assessed using a Wilcoxon and Kruskal Wallis test. Receiver operator curve analysis was used to determine the sensitivity and specificity for duration of clinical signs to predict the presence of hydromyelia.

**Results:**

Hydromyelia was identified at sites of IVDE in 84 of 91 dogs. An absence of hydromyelia was associated a with statistically longer duration of clinical signs (mean 73.1, IQR 76 days) when compared to cases with mild (mean 17.7, IQR 7.25 days, *P* = .006) or severe (mean 17.9, IQR 10.25 days, *P* = .006) hydromyelia. Duration of clinical signs <14 days was 78.6% sensitive and 85.7% specific for predicting the presence of hydromyelia.

**Conclusions and Clinical Importance:**

The MRI finding of hydromyelia might be a predictor of lesion chronicity in focal IVDE, helping to guide planning of hemilaminectomy surgery.

**Abbreviations**
CSA: cross‐sectional area
IVDE: intervertebral disc extrusion
MRI: magnetic resonance imaging
ROC: receiver operator curve

## 1 INTRODUCTION

Intervertebral disc extrusion (IVDE) and secondary compressive myelopathy is the most common cause for spinal cord injury in dogs,^1^, ^2^ representing 2.3% of all admissions into veterinary teaching hospitals.^3^ Extruded intervertebral disc material and hemorrhage causes attenuation of the spinal canal and variable degrees of spinal cord injury including compression, edema, necrosis, hemorrhage, and contusion.^4^ This spinal cord injury can cause profound neurological dysfunction that commonly results in the need for urgent decompressive laminectomy.

The mainstay for diagnosis of IVDE is magnetic resonance imaging (MRI). This modality has the contrast resolution to detect displacement of disc material, confirm the location of spinal cord compression and assess the degree of spinal cord injury. The MRI features of acute IVDE have been well documented^5^, ^6^ and multiple different variables have been assessed to identify MRI features that can predict neurological status and prognosis, including degree of cord compression, spinal cord hyperintensity and loss of subarachnoid signal.^7^, ^8^, ^9^ There is a lack of information describing the MRI appearance of more chronic lesions.^10^ This lack of predictive MRI features for lesion chronicity limits our ability to differentiate between sites of chronic and acute extrusion, a necessary part of surgical planning in dogs with IVDE.

The presence of hydromyelia is a potential indicator of IVDE chronicity. Hydromyelia is the distension of the ependymal lined central canal that communicates with the ventricular system.^11^ This is different from syringohydromyelia, where there is extension of the fluid through the ependymal lining and cavity formation within the spinal cord parenchyma, a feature that is not associated with IVDE histopathologically.^4^, ^12^ In the normal canine spinal cord, the central canal measures approximately 0.03 mm in diameter and is either undetectable or only minimally visible on sagittal and transverse T2‐weighted sequences; however, when hydromyelia is present the central canal distends and on good quality MRI images, becomes clearly visible centrally within the cord.^13^ The authors have identified that hydromyelia is a common feature of acute IVDE and likely to be related to the CSF flow disturbance caused by cord compression. However, at sites of chronic cord compression the authors have found that hydromyelia is often not present.

In this study, we investigate the presence of hydromyelia in the setting of focal thoracolumbar IVDE. We aim to characterize the MRI features of hydromyelia in a large cohort of dogs and statistically evaluate for any correlation between hydromyelia and duration of historic clinical signs, degree of cord compression and severity of neurological status.

## 2 MATERIALS AND METHODS

### 2.1 Case search

The picture archiving and communication system from the Cornell University Hospital for Animals was searched for cases that had undergone thoracolumbar MRI between October 2017 and September 2021 and had a diagnosis of a focal compressive IVDE in the T3‐L3 region. In addition, in order to increase the number of dogs with more chronic clinical signs, the Cornell University Hospital for Animals radiographic reports system was searched for cases with chronic thoracolumbar disc extrusions from January 2011 to October 2017.

### 2.2 Inclusion criteria

All included cases were required to have a single focal IVDE causing attenuation of the vertebral canal in the T3‐L3 region of the vertebral column. An extrusion was classified as displacement of intervertebral disc material to overlap the adjacent endplate or the displaced material had a height greater than its width. Cases needed to have a diagnostic quality sagittal T2‐weighted sequence with a transverse T2‐weighted sequence that spanned the site of IVDE and included the entirety of the abnormal region between sites of non‐compressed normal spinal cord. A complete medical history and a neurological examination before MRI were also required for inclusion.

### 2.3 Exclusion criteria

Cases were excluded if there was relevant artifact that impacted image quality or insufficient transverse slices to evaluate the spinal cord cranial and caudal to the site of extrusion. In order to keep the cohort as homogeneous as possible, cases were excluded if they had extruded material or hemorrhage that spanned >2 vertebral lengths, or spinal cord T2‐weighted hyperintensity or spinal cord swelling that spanned >2 vertebral body lengths cranial and caudal to the site of compression. In addition, cases were excluded if they had a history of a previous confirmed disc extrusion or hemilaminectomy, had more than a single site of IVDE or exhibited evidence of concurrent primary spinal cord disease.

### 2.4 Data collection

Data collected were: dog identification (number and name), signalment (age, breed, sex), date of MRI and site of IVDE. The duration of neurological signs observed before MRI was also recorded. The neurological examination was reviewed and a simple grading system utilized to score the neurological status of each case at the time of MRI [graded as the following; spinal pain only (1), paraparesis (2), paraplegia (3) or deep pain negative (4).

### 2.5 MRI evaluation

**FIGURE 1:**
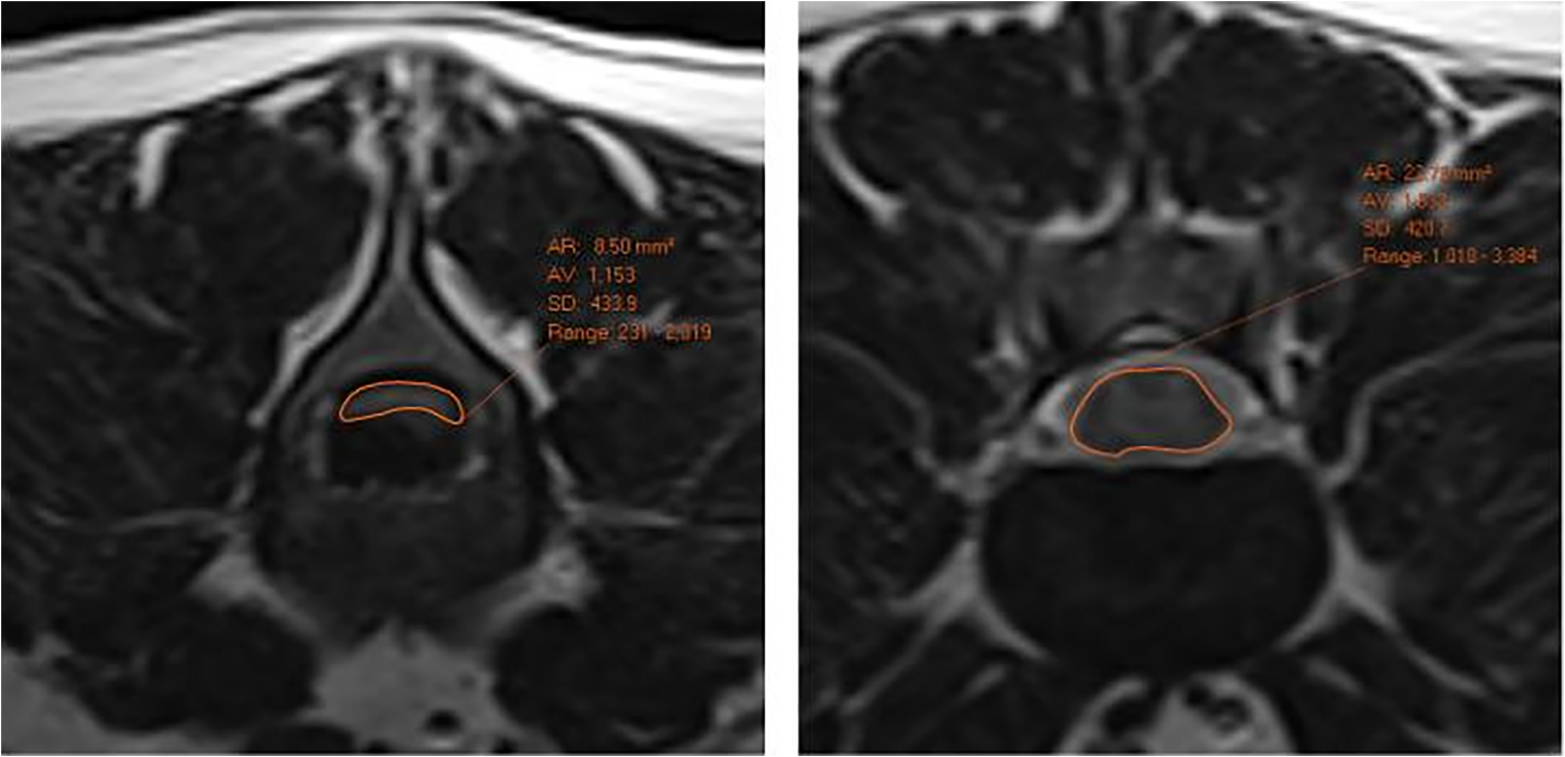
Demonstrates the cross‐sectional area measurements performed at the site of maximal compression (left) and 1 vertebral length caudal to the site of compression (right). The compression ratio was calculated by dividing the CSA of the cord at compression by the CSA of the normal cord

Imaging was performed on a 1.5‐T Toshiba Vantage Atlas MRI unit. All studies included sagittal and transverse T2‐weighted MRI sequences that spanned the site of IVDE (MRI sequence variables are documented in Table 1). Sagittal and transverse T2‐weighted MR images were independently blindly reviewed by an ECVDI‐certified radiologist and ACVR resident in training for the presence or absence of hydromyelia cranial and caudal to the site of spinal compression. The location of the hydromyelia in relation to the site of compression was recorded (only cranial, only caudal, or cranial and caudal to the site of cord compression). The degree of hydromyelia was graded as absent (0), mild (1; segmental central canal distension was mild, inconsistently visible on sequential transverse slices or short in length [<2 vertebral lengths]) and severe (2; segmental central canal distension clearly visible on sequential transverse slices or long in length [>2 vertebral lengths]) and cases were grouped according to these grades. Where disagreement was found, a consensus was formed. The cross‐sectional area (CSA) of spinal cord was measured by a single observer at the site of maximal compression and 1 vertebral length caudal to the site of compression, and a cord compression ratio calculated (CSA of the normal cord/CSA area of the compressed cord; Figure 1).^14^

**TABLE 1:** The magnetic resonance imaging (MRI) sequence variable ranges for the sagittal and transverse T2‐weighted sequences included in this study

MRI sequence	Echo time	Repetition time	NEX	Flip angle	Slice thickness	Matrix size	Echo train length	Phase encoding steps
Sagittal T2	94	2400 to 4050	1 to 2	90	2.8 to 4.0	612 to 794 × 480 to 496	13	507 to 650
Transverse T2	94	2700 to 5900	1 to 3	90	3.0 to 4.7	448 to 480 × 454 to 532	13	286 to 312

Abbreviations: NEX, Number of excitations.

### 2.6 Statistics

Histograms of the data were plotted to assess for normality. Depending on the normality of distribution, either an ANOVA with post hoc Tukeys or a Wilcoxon with Kruskal‐Wallis testing was applied to evaluate for statistically significant differences between groups with absent, mild, and severe hydromyelia for the continuous variables of; cord compression ratio, neurological severity grade and duration of clinical signs. In order to assess if duration of clinical signs could predict the presence or absence of hydromyelia on an MRI, a receiver operator curve (ROC) was generated; area under the curve was calculated and sensitivity and specificity determined for the most appropriate threshold.

## 3 RESULTS

### 3.1 Cohort signalment

Ninety‐one cases fitted our inclusion criteria. This cohort was composed of 54 males and 37 females with a mean age of 7.0 years (±3 years). The breed distribution of the cohort included Mixed breed (n = 30), Dachshund (n = 27), German Shepherd Dog (n = 5), Beagle (n = 4), Labrador Retriever (n = 3), Shih Tzu (n = 3), Coton De Tulear (n = 2), Cocker Spaniel (n = 2), French Bulldog (n = 2), Pembroke Welsh Corgi (n = 2), Pug (n = 2), Weimaraner (n = 2), Basset Hound (n = 1), Cairn Terrier (n = 1), Chihuahua (n = 1), English Mastiff (n = 1), Rat Terrier (n = 1), Rottweiler (n = 1), and Schnauzer (n = 1). All dogs underwent MRI and had a confirmed focal thoracolumbar IVDE in the T3‐L3 region. No dog underwent necropsy.

### 3.2 MRI examinations

**FIGURE 2:**
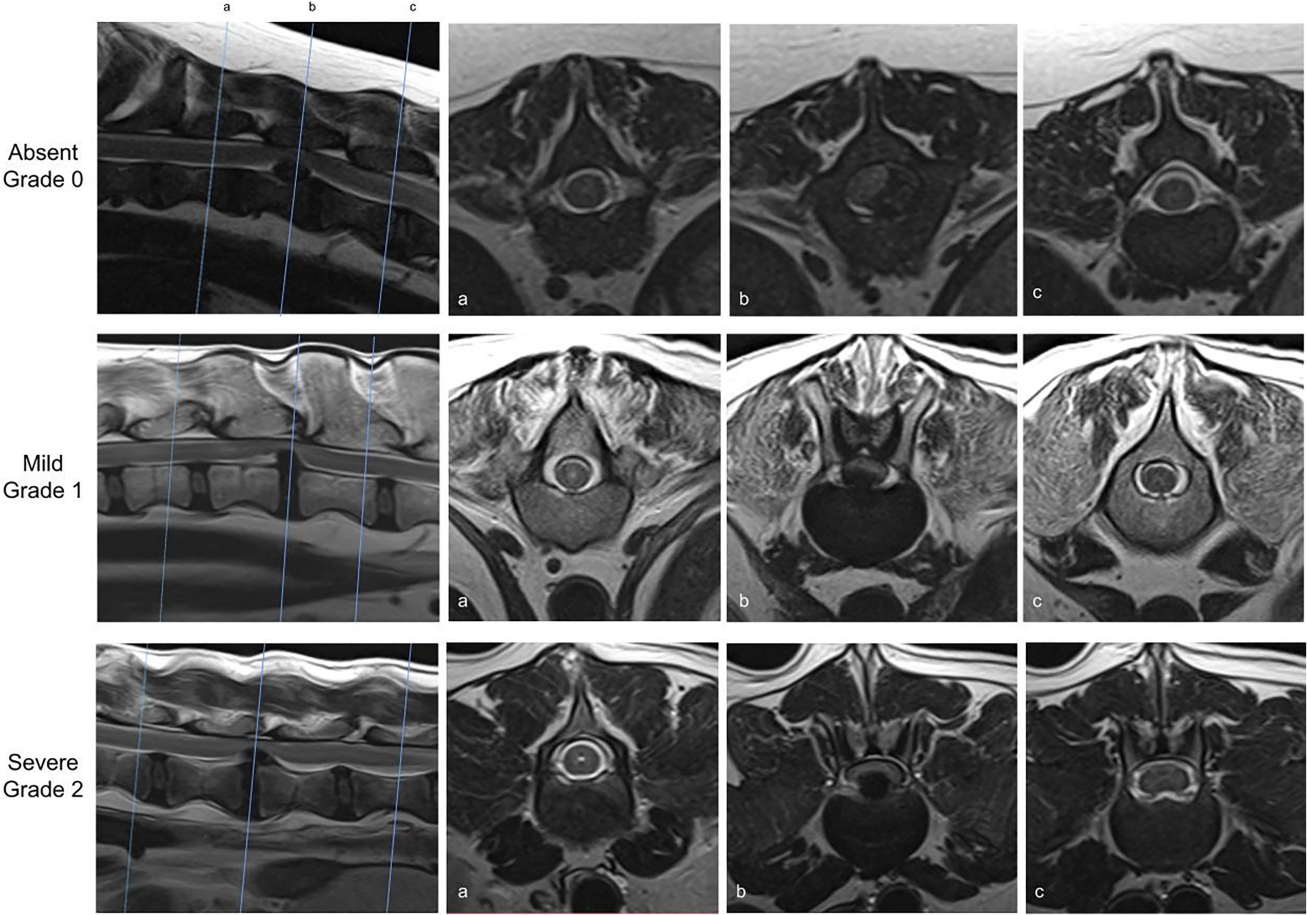
Sagittal and transverse T2‐weighted images. Demonstrates examples of absent (grade 0), mild (grade 1), and severe (grade 2) forms of hydromyelia

**FIGURE 3:**
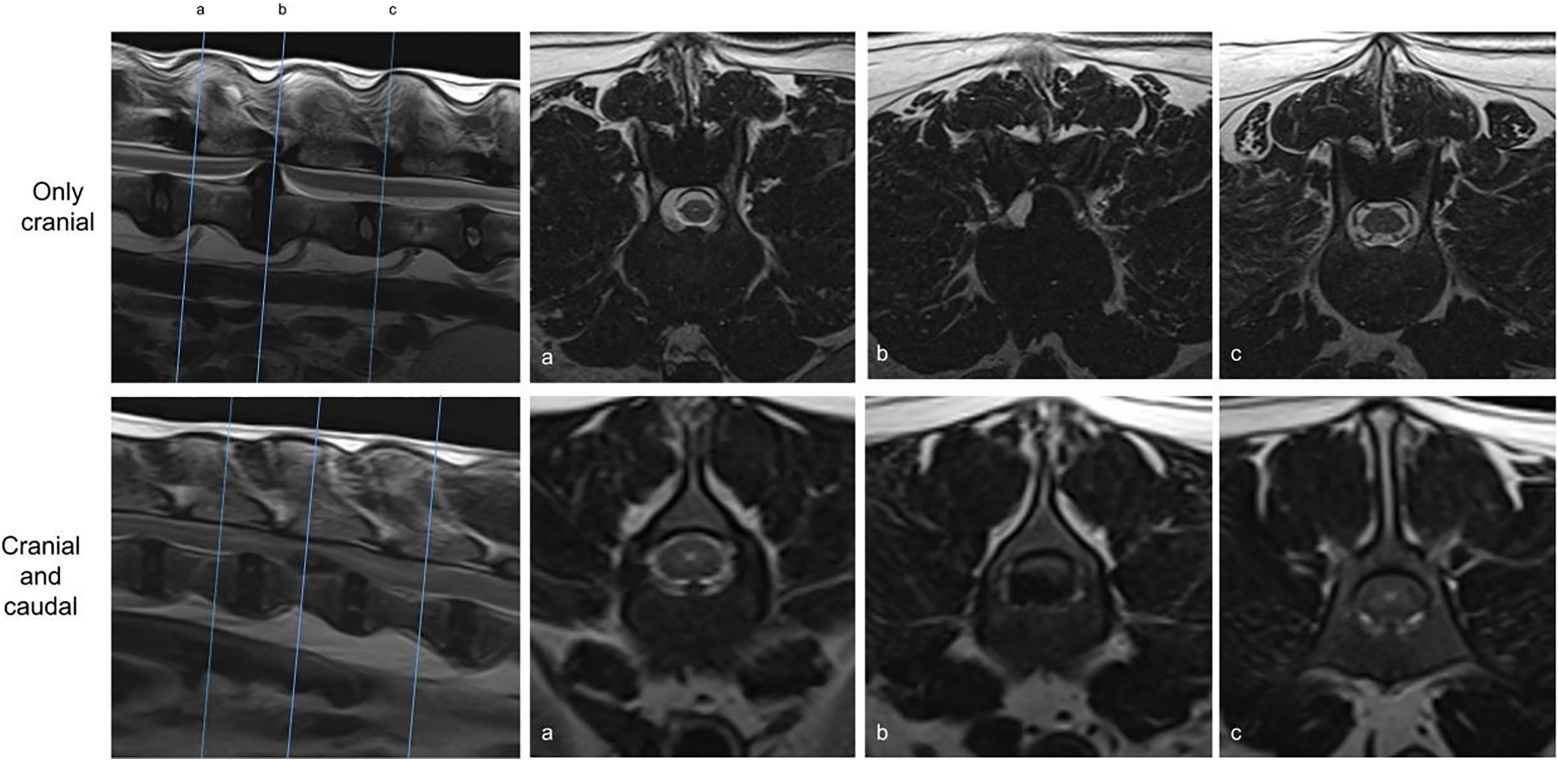
Sagittal and transverse T2‐weighted images. Demonstrates the types of hydromyelia in terms of location to the site of compression

The most common sites of IVDE were T12‐13 (n = 26), T13‐L1 (n = 25), and L1‐2 (n = 13), with other sites less commonly affected; L2‐3 (n = 8), L3‐4 (n = 8), T10‐11 (n = 1), T11‐12 (n = 7), T4‐5 (n = 1), T8‐9 (n = 1), and T9‐10 (n = 1). These sites of extrusion were associated with hydromyelia in 84 out of the 91 cases. The hydromyelia was graded as mild in 17 cases and severe in 57 cases (Figure 2). Hydromyelia was present cranial to the site of compression in 65 cases and was both cranial and caudal to the site of spinal cord compression in 19 cases. When hydromyelia was present both cranial and caudal to the site of compression, the length and severity of hydromyelia was always greater cranially when compared to caudally (Figure 3).

### 3.3 Analysis results

#### 3.3.1 Duration of clinical signs

**FIGURE 4:**
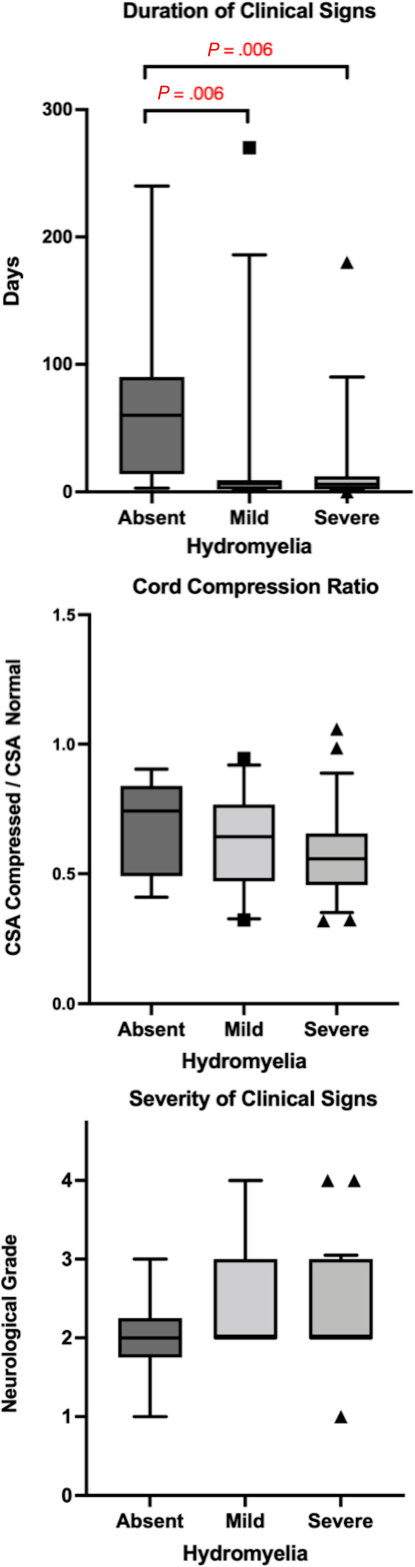
Box and whisker plots demonstrating the duration of clinical signs, cord compression ratio, and severity of clinical signs for each hydromyelia group (absent, mild, and severe)

**FIGURE 5:**
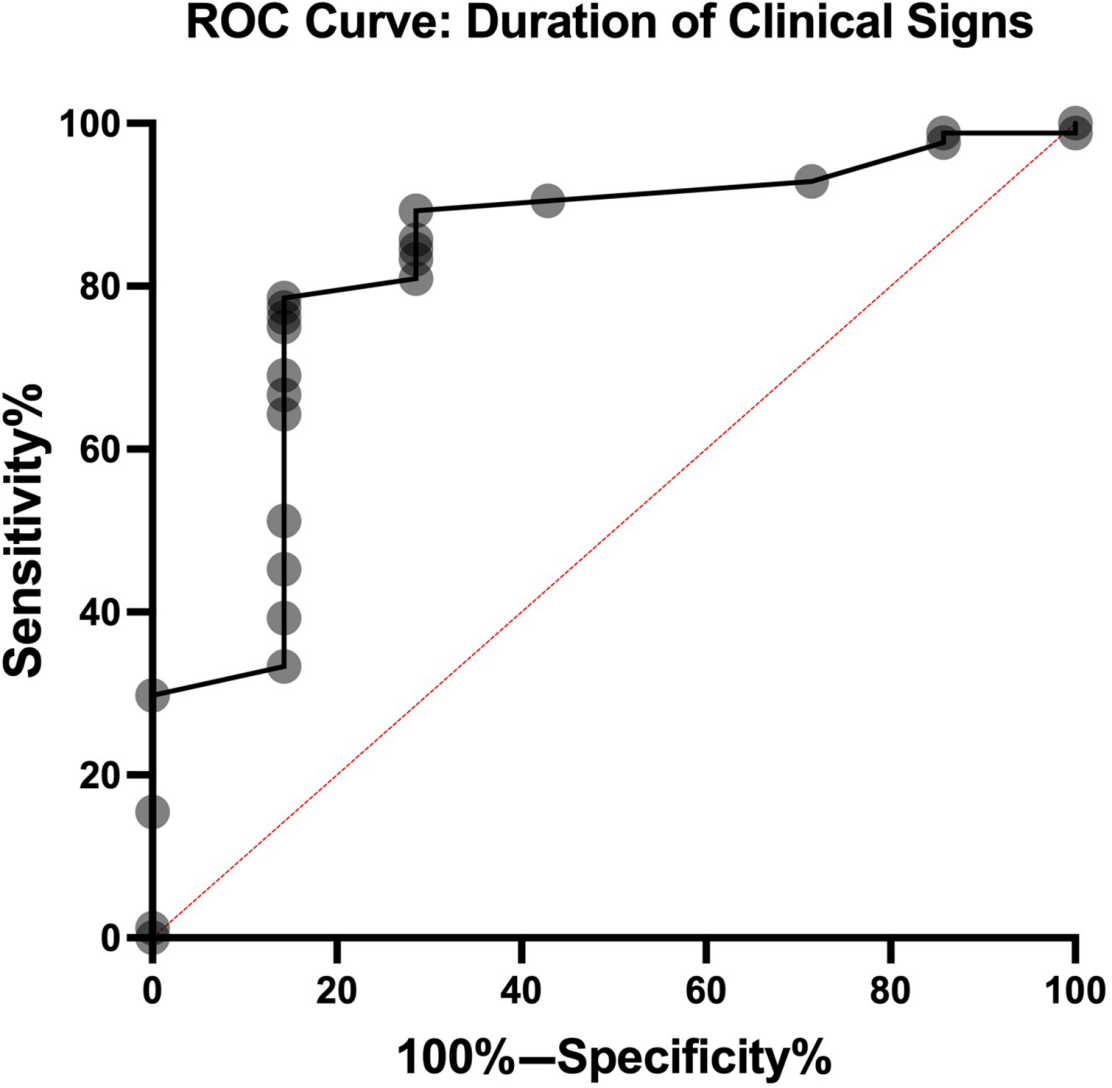
Demonstrates the receiver operator curve generated from the duration of clinical signs data. Sensitivity was plotted against specificity and provided an area under the curve value of 0.83. A threshold of 14 days provided a 78.6% sensitivity and 85.7% specificity for predicting the presence of hydromyelia on magnetic resonance imaging

The mean duration of clinical signs was 22.1 days (±45.3 days). When categorized according to clinical timeframe, 56 cases had a duration of clinical signs under 7 days in length, 22 cases had duration between 8 and 30 days, and 14 cases had a duration of greater than 30 days. The duration of clinical signs was statistically significantly longer in the absent hydromyelia group (mean 73.14, IQR 76 days) when compared to the mild (mean 17.73, IQR 7.25 days, *P* = .006) and severe hydromyelia groups (mean 17.87, IQR 10.25 days, *P* = .006). No statistically significant difference in duration was identified between mild and severe hydromyelia groups (Figure 4). When a ROC was plotted, the area under the curve was 0.83 and a ROC analysis identified that a duration of clinical signs <14 days was 78.6% (95% CI 0.098) sensitive and 85.7% (95% CI 0.140) specific for predicting the presence of hydromyelia on MRI (Figure 5).

#### 3.3.2 Degree of neurological dysfunction

Two dogs presented for spinal pain alone, 58 dogs presented with paraparesis, 27 presented with paraplegia, and 4 were deep pain negative. No statistically significant difference in neurological severity grade was identified between absent, mild, and severe hydromyelia groups (Figure 4).

#### 3.3.3 Degree of spinal cord compression

The spinal cord compression ratio was not statistically significantly different between cords without hydromyelia (0.69 ± 0.07), with mild hydromyelia (0.62 ± 0.03) and with severe hydromyelia (0.58 ± 0.02; Figure 4).

## 4 DISCUSSION

This investigation documents the presence of hydromyelia in association with focal thoracolumbar IVDE in a cohort of dogs. We found that the majority of dogs with focal IVDE exhibited hydromyelia either cranial or both cranial and caudal to the site of compression. The presence of hydromyelia, regardless of grade, was significantly associated with a shorter duration of presenting clinical signs, and more chronic IVDEs were less likely to exhibit this finding. These results suggest that the presence or absence of hydromyelia might help in the differentiation of acute versus chronic IVDE.

CSF moves along the spine in a cranial and caudal manner. However, the caudal movement of fluid is faster and shorter in duration than cranial movement. These alterations in velocity are associated with pressure fluctuations along the spinal cord.^15^, ^16^ Hydromyelia identified in this study was cranial to sites of spinal cord compression in the majority of cases and both cranial and caudal to sites of compression in a smaller subset. In addition, we identified both mild and severe forms of hydromyelia. These sites of compression are likely to have caused some level of obstruction to CSF flow. Geometric computational models have identified that such obstructions increased CSF flow velocities and pressure gradients.^17^ These alterations in CSF flow dynamics are likely to account for the variation in location and severity of hydromyelia observed in our study.

We made the presumption that the canal distension observed on MRI in this study is hydromyelia and not syringohydromyelia. Syrinx formation is a condition that should be readily visible on histopathology as cavitation within the spinal cord and, as it has not been described as a common component of IVDE histopathologically, is unlikely to be the cause for the MRI changes we describe in this study.^11^, ^12^ Central canal distension has also not been described on histopathology of IVDE. During removal of the spinal cord the pressures within the central canal will reduce, likely making the hydromyelia that we observe in‐vivo inapparent on histopathology. These similar pressure‐related changes should not influence the presence of a syrinx in the same way. Despite this reasoning, without histopathological confirmation of each case, the precise cause for the MRI changes described in this study cannot be completely confirmed.

Although hydromyelia has not been described in histopathology or MRI descriptions of IVDE, previous work on myelography of IVDE has observed positive contrast to fill the central canal in a subset of cases. These “canalograms” are observed as a fine (<1 mm diameter) centrally located line within the spinal cord adjacent to sites of spinal cord compression.^18^, ^19^ The central canal highlighted in these “canalograms” is enlarged when compared to what is described in normal dogs and therefore likely represent sites of hydromyelia.^13^, ^18^, ^19^ They also visually appear similar to the distended central canals described in this study.^18^, ^19^

MRI descriptions of IVDE have not identified hydromyelia as an imaging feature of IVDE.^5^, ^6^ This might be related to the fact that many of the original publications were performed on MRI units with older technology, limiting their spatial resolution and reducing the visibility of this subtle MRI finding.^5^, ^8^ Indeed, several recent publications, which have included figures of high quality MRIs of IVDE, clearly show the presence of hydromyelia cranial to sites of IVDE.^6^, ^9^ MRI quality is likely to impact the visibility of hydromyelia in IVDE, and that lower field MRIs and studies with poor in plane spatial resolution might not resolve this subtle MRI finding.

In the dogs included in this study, the incidence of hydromyelia within the spinal cord decreased as duration of clinical signs increased. This finding indicates that as IVDE‐associated cord compression becomes more chronic, there are changes to the spinal cord that cause hydromyelia to subside. An explanation for this is that overtime attenuation of the central canal and obstruction of CSF flow reduces. This is likely because overtime the acute inflammation, edema, hemorrhage and cord swelling associated with the acute extrusion event abates, and the continual chronic spinal cord compression causes loss of nerve cells, gliosis, and edema within the gray matter and demyelination and Wallerian degeneration in the white matter.^10^, ^20^, ^21^ On a larger scale, these processes result in progressive reduction in spinal cord volume at the site of persistent compression. This leads to progressive reduction in attenuation of the central canal and restoration of normal CSF flow. This course of events could explain why we are seeing subsidence of hydromyelia in more chronic IVDE.

Despite the need to identify MRI features that predict lesion chronicity, there is limited information available on the imaging features of chronic IVDE in dogs, with the majority of studies only documenting the chronic changes observed after decompressive surgery.^10^, ^22^ The gliosis, atrophy, and degeneration associated with chronic spinal cord compression in humans cause T2‐weighted hyperintense and T1‐weighted isointense intramedullary lesions on MRI.^20^, ^21^ These signal intensity changes have been described in chronic spinal cord compression secondary to cervical spondylomyelopathy in dogs.^23^, ^24^ These changes are only associated with spinal cord changes secondary to chronic, long‐term cord compression, and although they are used clinically to identify chronic compressive myelopathy secondary to IVDE, they are not useful for the detection of compressions that are yet to cause these advanced spinal cord lesions. In this study, hydromyelia was found to be a reliable indicator of acute IVDE <14 days in duration. As such, the absence of hydromyelia in the presence of IVDE could be used to help identify sites of more chronic IVDE that have yet to develop severe spinal cord atrophy and degeneration. In the clinical setting, despite relatively high sensitivities and specificities for the presence of hydromyelia to predict acute IVDE, cases of false positives and false negatives were identified and that this finding is not a perfect test. With this in mind, this MRI feature should be used as an aid and be married to the clinical history, neurological presentation, and examination to build the body of evidence toward which intervertebral disc is the most clinically relevant for the case.

Our study did not find an association between hydromyelia and the severity of clinical signs observed at MRI. These results could have been impacted by the cohort we tested. In order to limit heterogeneity within our cohort, the study had a strict inclusion criterion to only include cases of focal disc extrusion and associated myelopathy. This resulted in removal of cases where there was extensive cord compression or myelopathy (spanning >2 vertebral lengths). The removal of these more severe cases is likely to have impacted our results because spinal cord hyperintensity correlates to neurological severity in IVDE, and likely accounts for our cohort only including 4 dogs with deep pain negative paraplegia.^5^, ^6^, ^25^

Our study found that the severity of spinal cord compression was not significantly different between hydromyelia groups. This lack of association was surprising as higher degree of spinal cord compression is expected to disrupt CSF flow more and increase the incidence and severity of hydromyelia. However, our study design might have been a cause for this lack of statistical difference in our results as, although we included a total of 91 cases, only 7 of these cases did not exhibit hydromyelia. This low number could have reduced our power to detect statistically significant differences between groups for some of the variables evaluated, and future work could focus on evaluating a higher number IVDE cases that do not exhibit hydromyelia.

This study utilized a clinical data set evaluated retrospectively. As such, there was variability in the MRI sequence variables between subjects. This lack of standardization might have reduced our ability to visualize hydromyelia. In particular, slice thickness was variable in the sagittal plane sequences, potentially reducing our ability to detect hydromyelia on these sequences. For this reason, the transverse slices were used primarily for the detection of hydromyelia in all cases.

This study explored the clinical and MRI features of hydromyelia in dogs with focal thoracolumbar IVDE and compressive myelopathy. The findings identified in this paper suggest that hydromyelia might be an MRI feature of acute IVDE and its absence could be a predictor of more chronic of IVDEs. Being able to differentiate between acute and chronic IVDE at the time of MRI is an important component of the preoperative assessment for surgical planning in dogs with IVDE.

## CONFLICT OF INTEREST DECLARATION

Authors declare no conflict of interest.

## OFF‐LABEL ANTIMICROBIAL DECLARATION

Authors declare no off‐label use of antimicrobials.

## INSTITUTIONAL ANIMAL CARE AND USE COMMITTEE (IACUC) OR OTHER APPROVAL DECLARATION

Authors declare no IACUC or other approval was needed.

## HUMAN ETHICS APPROVAL DECLARATION

Authors declare human ethics approval was not needed for this study.
